# The Emerging Concern and Interest SARS-CoV-2 Variants

**DOI:** 10.3390/pathogens10060633

**Published:** 2021-05-21

**Authors:** Edyta Janik, Marcin Niemcewicz, Marcin Podogrocki, Ireneusz Majsterek, Michal Bijak

**Affiliations:** 1 Biohazard Prevention Centre, Faculty of Biology and Environmental Protection, University of Lodz, Pomorska 141/143, 90-236 Lodz, Poland; edyta.janik@unilodz.eu (E.J.); marcin.niemcewicz@biol.uni.lodz.pl (M.N.); marcin.podogrocki@biol.uni.lodz.pl (M.P.); 2 Department of Clinical Chemistry and Biochemistry, Medical University of Lodz, 90-419 Lodz, Poland; ireneusz.majsterek@umed.lodz.pl

**Keywords:** COVID-19, SARS-CoV-2, mutations, variants

## Abstract

The severe acute respiratory syndrome coronavirus 2 (SARS-CoV-2) responsible for coronavirus disease 2019 (COVID-19) was discovered in December 2019 in Wuhan, China. Since that time, the virus has spread around the world, which resulted in an announcement of the World Health Organization (WHO), dated in March 2020, that COVID-19 was a worldwide pandemic, and since then, the world has been struggling with this disease. SARS-CoV-2, similar to other RNA viruses, continually mutates, and new variants are appearing. Among large numbers of detected SARS-CoV-2 variants, only an insignificant amount of them are able to pose a risk to public health, as they are more contagious and cause more severe conditions. The emerged variants were classified by the Centers for Disease Control and Prevention (CDC) in collaboration with SARS-CoV-2 Interagency Group (SIG) according to strictly defined pattern. Variants were classified as variants of concern, variants of interest, and variants of high consequence. In the last few months, three variants of concern (B.1.1.7, B.1.351, and P.1) and four variants of interests (B.1.526, B.1.525, B.1.427/B.1.429, and P.2) were distinguished and are essential for close monitoring. This analysis summarizes the principal information concerning SARS-CoV-2 variants, such as their infectivity, severity, mutations, and immune susceptibility.

## 1. Introduction

Severe acute respiratory syndrome coronavirus 2 (SARS-CoV-2) is a particularly transmissible virus causing the infectious disease that appeared in late 2019 in China and led to the pandemic of the acute respiratory disease known as coronavirus disease 2019 (COVID-19) [1]. By mid-May 2021, the ongoing pandemic has led to almost 162 million confirmed cases and over 3.3 million deaths [2].

Mutations are an inherent feature of all viruses; however, RNA viruses present higher mutation rates than DNA viruses [3,4]. The mutability magnitude of RNA in comparison to some DNA viruses can exceed by up to five orders [5]. RNA viruses engage an intrinsically error-prone RNA polymerase in their replication process, and their genomes assemble mutations in each step of copying cycle. Furthermore, these cycles may occur within hours, ensuring diverse virus population generation within one infected host [6]. Coronaviruses (CoVs) cause less mutations than other RNA viruses due to enzyme possession, which excises erroneous mutagenic nucleotides incorporated by RNA polymerases. In consequence, they preserve a relatively elevated replication accuracy and virus transcription [7]. In majority of the cases, natural selection regulates the destiny of the newly created mutation [8,9]. Usually, mutations, which provide an opposed advantage with respect to transmission, viral replication, or immunity escape, will escalate in frequency, and consequently reduce the viral efficiency. Furthermore, they tend to be removed from the circulating virus community. Although, mutations’ frequency can also randomly increase or decrease [3,10]. RNA viruses show substantial potential to adapt to new habitats and hosts, and to withstand the impact of various selective pressures. The selective pressures against viruses include not only the host’s immune system and defense mechanisms, but also medicines such as antiviral drugs [11]. During the pandemic progression, viruses come across diverse host genetic variety and distinct cellular microenvironments. This can lead to increased probability of virus mutations, which influence their virulence, transmissibility, or pathogenesis [12,13].

The virus with one or more new mutations is referred as the original virus variant. Variants can differ among themselves by one mutation or many [14]. As viruses mutate constantly, new variants are expected to emerge over time. Sometimes new variants occur and disappear randomly and sometimes they occur and persist [15]. Numerous SARS-CoV-2 variants have already been documented globally during the COVID-19 pandemic [16]. To improve coordination among the National Institutes of Health (NIH), US Centers for Disease Control and Prevention (CDC), US Food and Drug Administration (FDA), Biomedical Advanced Research and Development Authority (BARDA), and Department of Defense (DoD), the Department of Health and Human Services (HHS) SARS-CoV-2 Interagency Group (SIG) was established. The aim of this group is to rapidly characterize the emerging variants and to monitor their possible influence on critical SARS-CoV-2 countermeasures, primarily diagnostics, therapeutics, and vaccines. The CDC, in collaboration with SIG, developed a classification system for SARS-CoV-2 variants based on the threat level they pose to the public health. Variants are classified as variants of concern, interest, and variants of high consequence [17]. Three variants, which have rapidly become dominant in several countries, have raised concerns as they possess mutations of interest and providing evidence of international spreading: B.1.1.7, B.1.351, and P.1 [18]. According to the CDC and World Health Organization (WHO) in a weekly epidemiological report, the variants of interests include B.1.526, B.1.525, B.1.427/B.1.429, and P.2 [17,19]. So far, there are no SARS-CoV-2 variants that are classified as variant of high consequence [17].

All variants share one specific mutation called D614G. The D614G mutation became the predominant globally circulating variant after its appearance in the early COVID-19 pandemic [20]. The D614G is a non-synonymous mutation resulting in a replacement of aspartic acid with glycine at position 614 of the virus’s spike (S) glycoprotein [21]. The D614G mutation can influence on SARS-CoV-2 infectivity. Korber and colleagues [8] have found that this mutation is associated with higher levels of viral RNA in the upper respiratory tract in patients, and this suggests that a higher viral load is responsible for higher infectivity. What is more, Zhang et al. [22] have showed that, the D614G mutation can enhance SARS-CoV-2 infectivity by increasing S protein incorporation into the virion. In one study, a relationship between the G614D mutation and higher fatality rates has been reported [23]. However, Korber and colleagues [8] did not find significant evidence of G614D mutation effects on disease severity.

A consistent nomenclature has not been established for the SARS-CoV-2 variants. The three main, generally used terminologies have been proposed (PANGO lineage, Nextstrain, and GISAID) and the first two of them are used in this manuscript. The Nextstrain nomenclature system is based on a large scale diversity of SARS-CoV-2 patterns and label clades that can persist for at least several months and have significant geographic spread [24]. The PANGO lineage nomenclature focuses on the epidemiological event, for example, on virus introduction into a distinct geographic area with evidence of onward spread [25].

The objective of this assessment is to describe and analyze the current classified SARS-CoV-2 variants. The authors focus mainly on the variants that cause concern, their characteristic mutations, and their consequences, as well as summarizing the latest research aiming to investigate their pathogenicity or transmissibility. In order to prepare the manuscript, the SCOPUS databases, PubMed, and Sage Journals were explored. The data from WHO, CDC, ECDC, FDA, Cov-leneages.org, and statista.com were also taken into account. In total, the manuscript consists of 75 of the most recent positions on this topic, including original research, reviews, and data from previously mentioned agencies. Identified applicable literature positions were hand searched using the following terms: COVID-19, SARS-CoV-2, SARS-CoV-2 mutations, SARS-CoV-2 variant, variant of concern (VOC), SARS-CoV-2 lineage, B.1.1.7, B.1.351, and P.1 SARS-CoV-2.

## 2. Variant of Concern

**Figure 1:**
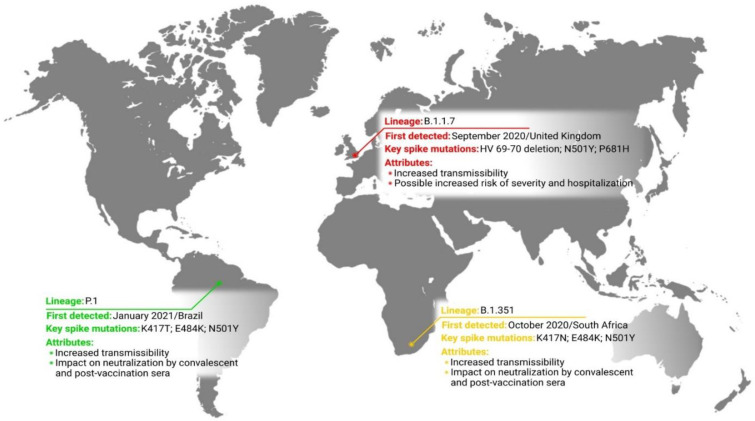
Current localization of SARS-CoV-2 variants of concern and their brief characteristics.

The variant of concern is defined when there is evidence of increased transmissibility, more severe disease (enlarged number of hospitalizations or death rate), significant reduction in antibodies’ (Abs) neutralization generated during former infection or vaccination, decreased treatment or vaccine efficacy, and error detection protocols [17]. Based on characteristics of variants of concern, different public health actions may be required (e.g., advancement of new diagnostics protocols or modification of vaccination or treatments strategies) in order to control spreading or to determine the testing effectiveness and potential vaccines and therapies against the variant [17,26]. Currently, there are three variants of concern that have emerged independently in different places and at different times and possess different mutations and attributes (Figure 1) [17].

### 2.1. Lineage B.1.1.7

The SARS-CoV-2 lineage B.1.1.7 (also referred as 20I/501Y.V1 or VOC-202012/1) was first recognized among COVID-19 patients in the south-east part of England in September 2020 [27]. In early December 2020, Public Health England (PHE) identified this variant as one of concern. Since then, the B.1.1.7 variant escalated to the rest of the UK territory, with 75% of all cases attributable to this variant by the end of December 2020 [28]. Currently, it is the dominant strain in the UK and its prevalence is growing in other countries and continents [29]. To date, more than 20,000 cases have been recorded in the United States (US), and it is present in almost all states [30]. According to the global reports investigating novel coronavirus haplotypes, the B.1.1.7 variant has been noted already in 114 countries [31].

This variant contains 17 mutations: 14 non-synonymous point mutations and 3 deletions (Table 1). Eight of them are placed in the gene that encodes the S protein and which mediates SARS-CoV-2 adhering and entrance into human cells [32]. Three mutations located in the S protein are of particular concern due to their likely phenotypic effect and are associated with enhanced infectivity and transmissibility. The HV 69–70 deletion appeared in many independent SARS-CoV-2 lineages, and is associated with immune escape in patients with immunodeficiency, and in vitro increases virus infectivity [33]. Immune escape is also contributed to by Y144 deletion. Wang et al. [34] found that the resistance of B.1.1.7 to most NTD-directed monoclonal antibodies (mAbs) is mostly conferred by ΔY144. N501Y mutation is a principal contact residue in the receptor-binding domain (RBD) and increases virus attachment predisposition to human angiotensin-converting enzyme 2 (hACE2) [32,35]. In silico protein–protein interface analysis has revealed that the increased infectivity of B.1.1.7 lineage is correlated with the increased interaction between the RBD Y501 mutant residue and ACE2 receptor, and in this strain, the elevated level is observed [36]. On the other hand, recognized mutation P681H directly adjoins to the spike furin cleavage site, a recognized location of importance for transmission and infection [37,38].

Graham et al. [39] explored whether the presence of the B.1.1.7 variant was associated with disease course, duration, necessity of hospitalization, and transmissibility among positively tested patients between September and December 2020. There was no relationship between the proportion of the circulated B.1.1.7 variant and severity of the disease. Furthermore, the proportion of people with prolonged symptom duration including demographic features (age, sex) and periodical variables (temperature, humidity) was also not observed. In addition, the proportion of individuals with an asymptomatic disease course did not significantly change as the incidence of B.1.1.7 increased. A different study [29] found that the B.1.1.7 variant was connected with a two-thirds elevated case fatality rate than the formerly circulating virus in the unvaccinated population. According to this analysis, the absolute risk of death within 28 days showed an increasing risk associated with older age and with comorbidities. Moreover, males were constantly at a higher risk of death than females. Nevertheless, those risk factors associated with poor non-B.1.1.7 variant outcomes appear to be similar to those with the B.1.1.7 variant. Davies and colleagues [33] combined numerous behavioral and epidemiological data with dynamic and statistical modeling, and calculated that the B.1.1.7 variant is 43–90% more transmissible than preexisting SARS-CoV-2 variants in England. As a result, existing control measures are less effective and stronger proactive interventions may need to be applied in order to obtain the similar level of control. However, on the basis of early population-level data, the authors were not able to identify a relationship between the occurrence of this variant and higher disease severity. In a recent English study performed from 1 September 2020 to 14 February 2021, the analysis of a dataset linking positive SARS-CoV-2 community tests and COVID-19 deaths showed that there is an association between B.1.1.7 infection and death rate. Results indicate an increase COVID-19 mortality related with the lineage B.1.1.7, and the authors estimated an approximately 61% (42–82%) higher hazard of death associated with the new variant [40]. Although underlying mechanisms of the B.1.1.7 variant spread are largely unknown, it seems to be connected with higher viral loads and extended persistence in the respiratory tract compared to other variants [41].

Wang et al. [34] have demonstrated that B.1.1.7 is refractory to neutralization by most of mAbs directed to the N-terminal domain (NTD) supersite, and moderately resistant to a few mAbs directed to the receptor-binding domain (RBD). However, it is not more resistant to convalescent plasma or vaccine sera. According to different research, the protective efficacy of existing SARS-CoV-2 vaccines and the neutralizing activity of vaccine sera against B.1.1.7 is highly intact, and this variant will not escape vaccine-mediated protection [42,43,44].

### 2.2. Lineage B.1.351

The SARS-CoV-2 lineage B.1.351 (also known as 501Y.V2) was first identified in October 2020 in South Africa—Eastern Cape Province and quickly spread throughout the country [45,46]. Detection of the B.1.351 variant occurred simultaneously with a rapid increase of confirmed COVID-19 cases in Zambia. Among the 23 individuals’ samples collected during one week in December 2020, 96% were the B.1.351 variant. Among 245 formerly sequenced genomes, none were from this lineage [45]. In the European Union/European Economic Area (EU/EEA), approximately 350 cases have been detected in 16 countries. Some cases reported in the EU/EEA were travel related; however, cases with no epidemiological link are increasingly reported. What is more, a large number of B.1.351 variant cases have been reported in Belgium and Austria [47]. According to the CDC, this variant has been found in 36 US states [30]. As stated, in global reports investigating novel coronavirus haplotypes, the B.1.35. variant has been already detected in 68 countries [48].

This variant is defined by multiple mutations (Table 2). Nine of them are present in the gene, which encodes the S protein. The gene has accumulated mutations within the RBD and the NTD [49]. The RBD is the major target of neutralizing antibodies (NAbs) induced by SARS-CoV-2 infection, with residual activity directed at the NTD [50]. Three of spike mutations are in the NTD (L18F, D80A, and D215G), three are at key residues in the RBD (N501Y, E484K, and K417N), and one is in loop 2 (A701V) [51]. Although variant B.1.351, similar to the variant B.1.1.7, also possesses the N501Y mutation in the S protein, variant B.1.351 arose independently from a different SARS-CoV-2 lineage [52].

The E484K and N501Y mutations are localized within the receptor-binding motif (RBM), which is the main functional and structures the interface with the hACE2 receptor [51]. The N501Y mutation is suggested to be associated with increased transmissibility [45].

Deep mutational scanning has shown that E484K reasonably augments the binding affinity of the ACE2 receptor [53]. What is more, E484K, K417N, and mutations in the NTD have become a serious global concern as they have been shown to be associated with NAbs escape [54]. Additionally, L242/244 deletion located at the NTD shows a loss of NAbs’ binding ability [34,55]. Cele et al. [50] presented the data suggesting that B.1.351 is able to escape the neutralizing antibody response induced by natural infection caused by earlier variants. These results raised concerns about the potential to reduce protection against reinfection and the effectivity of vaccines elaborated to target the S protein from earlier SARS-CoV-2 variants. In addition, Wang et al. [34] showed that B.1.351 is resistant to neutralization by mAbs aimed to the NTD supersite. B.1.351 is also resistant to the main group of potent mAbs that targets the RBM, including three regimens approved for emergency use. What is more, this variant is markedly resistant to neutralization by vaccine sera and convalescent plasma. Those findings present the challenge for mAbs therapy and may jeopardized the protective efficacy of existing vaccines. Currently, there is no evidence definitively suggesting that the B.1.351 variant has any influence on COVID-19 course severity [46,47].

### 2.3. Lineage P.1

The new lineage, named P.1 (descendent of B.1.1.28) was detected in Manaus, Brazil in January 2021 [56]. Cases of P.1 variant infection were observed among travelers arriving in Japan from Brazil on January 2021 [57]. The first case of the SARS-CoV-2 P.1 variant in the US was identified in Minnesota resident with travel history to the southeastern part of Brazil within the 14 days before symptom onset [58]. Currently, almost 500 cases of this variant have been reported in the US and it is present in 31 states [30]. In the EU/EEA, approximately 30 cases have been discovered in countries such as France, Italy, Germany, the Netherlands, and Spain. The variant’s ongoing community transmission in the EU/EEA has not been detected yet, but this cannot be excluded [47]. According to the global reports investigating novel coronavirus haplotypes, the P.1 variant has been already detected in 37 countries [59].

The preliminary phylogenetic study has shown that P.1 lineage carries 17 mutations including 3 deletions, 4 synonymous mutations, and 4 nucleotide insertions in comparison to its immediate ancestor (B.1.1.28) [56]. Additionally, 10 of the 17 mutations are localized in the virus S protein (L18F, T20N, P26S, D138Y, R190S, K417T, E484K, N501Y, H655Y, T1027I). Three key mutations are concentrated in the RBD S protein (K417T, E484K, and N501Y). The K417T and E484K mutations interact with hACE2, while E484K is present in the loop region outside the direct hACE2 interface [60]. Thus far, little is known about P.1 transmissibility, but it shares with B.1.325 some independently acquired mutations (K417NT, E484K, N501Y) and one with B.1.1.7 (N501Y), which seems to be linked with increased transmissibility [61].

Wang et al. [62] showed that P.1 is resistant to neutralization by various RBD-directed mAbs. Among them there are three with emergency use authorization, REGN10987 (imdevimab), REGN10933 (casirivimab), and LY-CoV555 (bamlanivimab). The resistance mechanism is related to possession of the E484K mutation. Furthermore, both vaccine sera and convalescent plasma showed a notable loss of neutralizing activity against P.1; however, the reduction is not so significant in comparison to the B.1.351 variant [34]. In a different study [63] conducted in February 2020 in Brazil, the COVID-19 immune plasma of convalescent blood donors had a 6-fold reduced neutralizing capacity against P.1 than against the B-lineage. In addition, five months later after booster immunization with CoronaVac (Chinese mRNA vaccine approved for emergency use in Brazil, Colombia, Mexico, China, Indonesia, and Turkey), plasma from vaccinated people failed to efficiently neutralize P.1 variant strains. This variant was also the cause of reinfection, which may be an outcome of limited and transient protective immunity generated by primary infection. Naveca and colleagues [64] reported the first confirmed case of P.1 lineage reinfection in a 29-year-old, immunocompetent woman in Brazil, who was an Amazonas state resident infected with the B.1 lineage approximately nine months before. The patient had equally average symptoms during both episodes and a larger viral load in nasopharyngeal and pharyngeal samples collected during reinfection compared to the primary infection samples. So far, nothing is known about the probable changes in the severity of infection in P.1 variant-infected individuals [47]. A brief summary of current variants of concern is presented in Table 3.

## 3. Variant of Interest

The SARS-CoV-2 variant of interest is when the variant presents phenotypic changes (changes in the virulence, antigenicity, and epidemiology, as well as changes that have or conceivably have a negative effect on available diagnostics protocols, vaccines, therapeutics, or public health and social measures) compared to the reference isolate [26]. The variant of interest possesses mutations, which stimulate amino acid changes related to possible or established phenotypic implications. Moreover, the variant has been recognized as a causative agent of community transmission/multiple COVID-19 cases/clusters [17,26].

Based on the detailed assessment, various SARS-CoV-2 variants have been classified as variants of interests [17,19,65,66]. Table 4 summarizes assessed and designated variants of interests as of 29 March 2021.

## 4. Variant of High Consequence

According to existing classification, a mutation causing the variant of high consequence is when there is a comprehensible proof that efficacy of prevention measures or medical countermeasures taken (such as vaccines, antiviral drugs, and mAbs) are significantly decreased in comparison to previously circulating variants. It also causes more severe disease course and importantly enlarges the number of hospitalizations necessary. Currently, none of the variants meets this criteria [17].

## 5. Mutations Impact on SARS-CoV-2 Diagnostics/Detection Protocols

The current standard for SARS-CoV-2 diagnostic is based on a molecular test of the reverse transcription quantitative real-time polymerase chain reaction (qRT-PCR). In order to detect the viral RNA in respiratory samples like nasopharyngeal swabs or bronchial aspirate are taken. Until now, a number of qRT-PCR assays have been developed [67]. Since its transmission to humans, SARS-CoV-2 has undergone adaptive evolution, resulting in genetic variation, which can be challenging, especially in molecular diagnostic protocol that identifies SARS-CoV-2 by targeting multiple positions in the viral genome [68]. The qRT-PCR oligonucleotides bind to small ~20 bp regions, and mutations in these targets can reduce efficient amplification or probe binding, thereby generating false negative results. Unlike other RNA viruses, CoVs have a moderate mutation rate due their ability to perform RNA correction. However, given the large number of ongoing transmission chains, it is reasonable to monitor the integrity of the qRT-PCR assays [69,70]. Ziegler et al. [71] reported that the single nucleotide polymorphism (SNP) in the N gene of SARS-CoV-2 from patients can interfere the detection process in widely used commercial assays. Although the virus is still detected by the other probes, this underscores the need of targeting at least two independent virus key regions for reliable detection. A different study has reported that the identification of C-to-U transition at position 26,340 of the viral genome is related with failure of the cobas SARS-CoV-2 E gene qRT-PCR, and it was observed in eight tested patients. As the cobas SARS-CoV-2 assay targets two genome positions, the patients carrying this variant were still SARS-CoV-2 positive [69]. In addition to molecular tests, antigen tests are widely used, and this type of assay detects the presence of viral antigens such as parts of the viral S protein. As molecular tests, they also can be affected by viral mutations [67]. While performing the validation study of the Abbott Panbio6™ COVID-19 Ag assay, it was noticed that some swab samples failed to generate a positive result despite the high viral load on RT-PCR tests. Sequencing analysis has shown discordant results and antigen tests revealed the presence of multiple disruptive amino-acid substitutions in the N antigen clustered in a region known to contain an immunodominant epitope. A part of the variants, undetected by the antigen assays, contained the mutations A376T coupled to M241I. What is more, the mutations sequences were overrepresented in negative antigen tests and RT-PCR-positive samples [72]. In order to avoid false diagnoses, the FDA recommendations to health care providers and clinical laboratory staff who use SARS-CoV-2 tests is to consider providing the negative results as a part of combination of test, patient history, clinical observations, and epidemiological information. Furthermore, in case of uncertainty after receiving a negative test result, it is recommended to repeat the test with a different European University Association (EUA)- or FDA-authorized molecular diagnostic test (with different genetic targets) if SARS-CoV-2 is still suspected [73].

## 6. Conclusions and Perspectives

Over the past few months, the emergence of new virus variants has become a new crucial point of interest in the ongoing COVID-19 pandemic. Many variants of SARS-CoV-2 have been discovered; however, the variants that have been recently identified in the UK, South Africa, and Brazil because of their features—increased transmissibility, evading immune responses triggered by previous infections, vaccine efficacy, escalating disease severity, and hospitalization necessity—are concern variants. Furthermore, there are opinions relating to the British mutation, which is completely different from the origin and more contagious and deadlier, that the existing pandemic should be renamed the COVID-20 outbreak. On the other hand, a recently discovered Israel P681H variant, which was previously identified in Nigeria and Hawaii, has not been associated with a higher infectious rate [74]. Further investigation is needed to explain this phenomenon. The possible explanation is the fast Israeli vaccination campaign, which resulted in the administration of approximately 116 doses of the COVID-19 vaccine per 100 people (data as of 31 March 2021) [75] and, thus, positively influenced and lowered the infectious rate. The scientific community focuses on better understanding all existing and possible variants in order to be able to evaluate their impact on vaccine efficacy, predict their development and spreading, and implement a specific treatment and management plan. Vaccination is a key tool in the ongoing battle against COVID-19 and should not be postponed because of concerns of newly appearing SARS-CoV-2 mutations. The most direct way to combat emerging variants is also to rapidly vaccinate as many people as possible using currently available and authorized vaccines. It is recommended that the vaccination strategy should continue, even in cases where vaccines may be slightly less effective against certain variants of the virus. It is expected that as more people receive their vaccine, the virus circulation will decrease and this will lead to the appearance of fewer mutations. It is therefore important to ensure equal access to COVID-19 vaccines and also to focus on the reposition or discovery of an efficient drug against SARS-CoV-2. Until that moment, the existing vaccines are the only valuable tool in the battle with SARS-CoV-2 and its variants. In the near future it is expected that newly elaborated vaccines will also directly target the existing and emergent variants of SARS-CoV-2.

## Figures and Tables

**Table 1:** Mutations defining the B.1.1.7 lineage with emphasis on mutations of particular concern [32].

Gene	Nucleotide	Amino Acid
ORF1ab	C3267T	T1001I
	C5388A	A1708D
	T6954C	I2230T
	11288–11296 deletion	SGF 3675–3677 deletion
Spike	21765–21770 deletion	HV 69–70 deletion (mutation of concern)
	21991–21993 deletion	Y144 deletion
	A23063T	N501Y (mutation of concern)
	C23271A	A570D
	C23604A	P681H (mutation of concern)
	C23709T	T716I
	T24506G	S982A
	G24914C	D1118H
ORF8	C27972T	Q27stop
	G28048T	R52I
	A28111G	Y73C
N	28280 GAT- > CTA	D3L
	C28977T	S235F

**Table 2:** A summary of non-synonymous lineage-defining mutations in the B.1.351 lineage [51].

Gene	Nucleotide	Amino Acid
ORF1ab	C1059T	T265I
	G5230T	K1655N
	C8660T	H2799Y
	C8964T	S2900L
	A10323G	K3353R
	G13843T	D4527Y
	C17999T	T5912I
Spike	C21614T	L18F
	A21801C	D80A
	A2206G	D215G
	22286-22294 deletion	L242_244L deletion
	G22299T	R246I
	G22813T	K417N
	G23012A	E484K
	A23063T	N501Y
	C23664T	A701V
ORF3a	G25563T	Q57H
	C25904T	S71L
E	C26456T	P71L
N	C28887T	T205I

**Table 3:** A summary of emerging information regarding variants of concern.

Characteristic	B.1.1.7	B.1.351	P.1
Characteristic spike mutations	HV 69/70 deletion, Y144 deletion, N501Y, A570D, D614G, P681H, T716I, S982A, D1118H	L18F, D80A, D215G, L242_244L deletion, R246I, K417N, E484K, N501Y, D614G, A701V	L18F, T20N, P26S, D138Y, R190S, K417T, E484K, N501Y, D614G H655Y, T1027I, V1176F
Transmissibility	Increased transmissibility (43% to 90% more transmissible than previously circulating variants).	Suggested increased transmissibility (50% more transmissible than the previously circulating variants).	Increased, 1.7–2.4-fold more transmissible than previous circulating variants
Severity	Possible increased risk of severity and mortality of illness.	No significant evidence of an impact on COVID-19 course severity or mortality.	No significant evidence of an impact on COVID-19 course severity or mortality.
Immune susceptibility	Refractory or moderately resistant to neutralization by monoclonal antibodies (mAbs). Not resistant to neutralization by convalescent plasma and vaccine sera.	Potential increased risk of reinfection. Resistant to neutralization by mAbs. Markedly resistant to neutralization by convalescent plasma and vaccine sera.	Potential increased risk of reinfection. Resistant to neutralization by mAbs. Notable loss of neutralizing activity by convalescent plasma and vaccine sera.
References	[32,33,34,39,42,43,44]	[45,46,47,51]	[47,60,62,63,64]

**Table 4:** Examples and characterization of variants of interests.

Name (PANGO Lineage)	Next Strain Clade	First Detected	Key Spike Mutations	Characteristic
B.1.526	20C	United States (November 2020)	T95I, D253G, L5F, S477N, E484K, D614G, A701V	Potential depletion in neutralization by convalescent and post-vaccination sera or monoclonal antibody treatments
B.1.525	20C	United States (December 2020)	H69-V70 deletion, Y144 deletion, Q52R; E484K, Q677H; D614G, F888L	Potential depletion in neutralization by convalescent and post-vaccination sera or monoclonal antibody treatments
B.1.427/B.1.429	20C/S:452R	United States (June 2020)	L452R, W152C, S13I, D614G	Decrease in neutralization using convalescent and post-vaccination sera, ~20% increased transmissibility
P.2	20J	Brazil (April 2020)	L18F, T20N, P26S, F157L, E484K, D614G, S929I, V1176F	Potential depletion in neutralization by convalescent and post-vaccination sera or monoclonal antibody treatments
